# An individual perspective on psychological safety: The role of basic need satisfaction and self-compassion

**DOI:** 10.3389/fpsyg.2022.920908

**Published:** 2022-08-18

**Authors:** Lies Wouters-Soomers, Joris Van Ruysseveldt, Arjan E. R. Bos, Nele Jacobs

**Affiliations:** Faculty of Psychology, Open University of the Netherlands, Heerlen, Netherlands

**Keywords:** psychological safety, self-compassion, basic need satisfaction, positive affect (PA), positive relations with others

## Abstract

Psychological safety is important for the well-being and productivity of people in the workplace. Psychological safety becomes even more important and even more difficult to maintain in times of uncertainty. Previous research mainly focused on the influence of and on interpersonal relationships. This study applies an individual perspective by investigating what is needed on an individual level in order to build psychological safety. The expectation was that self-compassion induces an individual to experience higher positive affect, and this advances the development of positive relations and social acceptance. Moreover, we assumed that the mediation of the relationship between self-compassion and positive relations and social acceptance by positive affect is moderated by the level of basic need satisfaction. Participants (*N* = 560) from the Netherlands and Belgium completed an online questionnaire about their level of self-compassion, basic need satisfaction, positive affect and positive relations and social acceptance. Using hierarchical regression analyses for moderated mediation analysis, results showed that self-compassion and positive affect had a significant positive effect on positive relations and social acceptance. Positive affect significantly mediated the relationship between self-compassion and positive relations and social acceptance, when basic need satisfaction was low, but not when basic need satisfaction was high. Our research showed that individuals need either their basic needs satisfied or self-compassion so they can build the high-quality relations needed to stimulate psychological safety. This finding shifts attention from the dyadic relationship to the individual and highlights important factors at the individual level which advance the development of high-quality relationships with others.

## Introduction

In times of significant organizational or environmental change the potential for anxiety is increased because people must take action without knowing whether things will work out as expected (Edmondson, 2002). Psychological safety connects changeable workplaces to the health, resilience, and well-being of individuals and teams (Shain et al., 2012). Lakhan et al. (2020) discovered that anxiety, stress, and psychological distress increased during the pandemic, making it more necessary for organizations to invest in ways to support their employees through this increased level of stress. According to Hebles et al. (2022) “By feeling safe in the work environment and not exposed to inter-personal risks, workers can feel less stress and reduce the emotional and cognitive consequences it brings. In particular, psychological safety can be an important mechanism to reduce stress by creating a climate of trust and risk-free communication.” Besides from the obvious need for psychological safety to deal with changeable workplaces and stress, a meta-analytic review by Frazier et al. (2017) also showed the effect of psychological safety on the performance of teams. Psychological safety was significantly linked to task performance, information sharing, commitment, learning behavior and creativity. Despite the convincing evidence of the benefits of psychological safety and its necessity in times of a pandemic, there are not too many organizations that offer a psychologically safe environment. According to Edmondson (2021): “More often than not, even though the organization lacks a toxic environment, people may still shy away from the interpersonal risks necessary to making progress on the transformative strategies the market environment demands.” A lot of research points toward the importance of high-quality relationships to build psychological safety (Carmeli et al., 2009; Frazier et al., 2017; Newman et al., 2017). And although there is much research into the benefits of psychological safety and why it is necessary, there is little known about why there is still such a lack in psychological safety. This research aims at showing that people need to feel safe themselves first in order to be able to start making the connections with the people that are needed to build a psychologically safe team.

### Psychological safety

Professors Schein and Bennis (1965) introduced the concept of psychological safety. They argued that psychological safety is essential in order to make people feel secure and capable of changing their behavior in response to shifting organizational challenges. The most used definition in research was introduced by Edmondson: “Psychological safety is a shared belief from the team members that the team is safe for interpersonal risk-taking.” (Edmondson, 1999, p. 354). She considers psychological safety to be a climate with the focus on productive discussion that makes it possible to discover and prevent problems in time and to pursue shared goals because team members feel less need to focus on self-preservation (Edmondson, 1999). “In psychologically safe environments, people believe that if they make mistakes others will not penalize or think less of them for it. They also believe that others will not resent or penalize them for asking help, information or feedback” (Edmondson, 2002, p. 5). To create psychological safety though, one needs to be able to find out how the members of their team think and feel (May et al., 2004). However findings from Buruck et al. (2014) indicate that acute psychosocial stress might impair empathic processes. This suggests that in order to make the connection to others to build psychological safety, one has to feel safe themselves first. To understand how, it is important to realize what makes an individual feel safe. According to Self-Determination Theory, individuals feel safe when their basic psychological needs are being satisfied (Ryan and Deci, 2000).

### Basic need satisfaction

Ryan and Deci (2000) formulated the Self-Determination Theory, explaining that everyone has three basic psychological needs: the need for autonomy, connectedness, and competence. Self-Determination Theory assumes that people are naturally inclined toward intrinsic motivation and the integration of goals. Individuals can also perceive their psychological needs to be actively undermined by others, what is called “need thwarting.” Autonomy-thwarting or controlling behaviors include using rewards, using intimidating language, making demands without providing a rationale, using conditional regard, and using excessive personal control (Bartholomew et al., 2009). Competence-thwarting behaviors consist of emphasizing others’ faults, discouraging people from trying difficult tasks, sending the message that someone is incompetent, and doubting their capacity to improve (Sheldon and Filak, 2008). Relatedness-thwarting behaviors include being distant with others, not connecting emotionally, excluding them, not listening, and not being available when needed (Sheldon and Filak, 2008). The behaviors that are described as need thwarting behaviors are exactly those that threaten the psychologically safe environment. When a person believes that others will not penalize them for asking help, they believe that others will not exclude them (relatedness-thwarting), will not doubt their capacity to improve (competence-thwarting), or will not use intimidating language toward them (autonomy-thwarting). So to make sure that the team is safer for interpersonal risk-taking, one needs to believe others will not “thwart” ones basic needs. When individuals do experience need-thwarting, they are likely to experience maladaptive outcomes like burnout, depression, negative affect, and physical symptoms (Bartholomew et al., 2011). This means that when a person’s needs are being thwarted, they build a psychological unsafe environment. Stanley et al. (2021) did a meta-analysis investigating basic need satisfaction and positive affect. They included 16 studies and found that higher positive affect was significantly associated with greater autonomy satisfaction, competence satisfaction, and relatedness satisfaction. When an individual’s basic needs are satisfied, this individual feels safe and experiences positive affect. For a person to have their needs satisfied, several studies point toward self-compassion. Findings from Svendsen et al. (2016) suggested that self-compassionate individuals are able to physiologically adapt emotional responses to stressful events with more flexibility. In addition, self-compassion training appears to reduce defensiveness in the face of social threat (Arch et al., 2014). Therefore, the current study aims at showing that when basic needs are not being satisfied, self-compassion is needed in order to build the high-quality relationships needed to build psychological safety.

### Self-compassion

Self-compassion is broadly described as treating oneself with kindness and concern when one is experiencing negative life events (Neff, 2003). Specifically, self-compassion is defined as being composed of three components: self-kindness (i.e., being kind toward oneself when encountering pain and shortcomings), common humanity (i.e., considering personal suffering as part of the shared human experience), and mindfulness (holding painful thoughts and feelings in mindful awareness without avoiding or exaggerating them). In general, literature has shown that self-compassion is positively associated with emotional well-being (Neff et al., 2007; Zessin et al., 2015), while negatively associated with the experience of life stress and negative affect (Ying and Han, 2009; Diedrich et al., 2014; Zhang et al., 2016). A systematic review shows that self-compassion training has a positive effect on work-related well-being (Kotera and Van Gordon, 2021). Moreover self-compassion has been shown to have a positive relation with basic needs satisfaction (Ghorbani et al., 2012). Moè and Katz (2020) showed that the higher the teacher self-compassion, the more they perceive their needs met. When individuals experience need-thwarting, they can use self-compassion to satisfy their basic needs so they no longer feel threatened. For example, when individuals experience their autonomy to be under threat, they can use self-compassion to hold this painful thought without avoiding or exaggerating it. They can consider this personal suffering as part of the human experience and offer themselves supportive words. Based on this line of reasoning, one could speculate that when self-compassion is used to satisfy individuals’ basic needs, when their basic needs were originally threatened, they will experience positive affect once those needs are met. This suggests that basic need satisfaction moderates the relation between self-compassion and positive affect.

### Positive affect

Positive affect refers to a positive emotional state. Davidson has found that positive emotional reactions lead to more activity in the left frontal lobe which leads people to engaging behaviors (Davidson et al., 1990). Rigoni et al. (2015) observed that positive affect impacted the time of a conscious intention to act and conclude that their observations “fit nicely with the idea that positive emotional states broadens selective attention.” Indeed the broaden and build theory of Fredrickson (2001) suggests that when individuals experience a positive emotion, they open their mind and connect more to other people. This could mean that in order to be interested in another person and empathize with them, one needs a positive emotion. An individual whose basic needs are being threatened could use self-compassion to create that positive emotion that is needed to connect to other people and build positive relations.

### Positive relations and social acceptance

Lamers et al. (2011) refer to positive relations when a person is interested in the well-being of others and is capable of empathizing with others. Carmeli et al. (2009) found in their research a strong relation between experience of high-quality relationships and psychological safety. While positive relations refer to being interested in others, social acceptance means that other people signal that they wish to include you in their groups and relationships (Leary, 2010). Moore et al. (2018) conclude in their review that positive affect does not just result from good relationships but can also cause them. Positive affect and close relationships seem to be reciprocally linked across the life span (Ramsey and Gentzler, 2015). The same goes for self-compassion. “Self-compassion is associated with a wide variety of close interpersonal relationship benefits. These associations may be complex and bidirectional, such that positive social relationships promote self-compassion, while self-compassion promotes relational and emotional well-being.” (Lathren et al., 2021, p. 1078). In this study we look at how self-compassion might lead to positive relations, through positive affect.

The current study is the start of a greater research project on how to build a psychologically safe environment. The focus in this study is on what is needed to make the connection to other people in order to start building that psychological safety.

The hypotheses in this research are:

**Hypothesis 1:** Self-Compassion is positively related to positive affect.

**Hypothesis 2:** The relation between Self-Compassion and positive relations and social acceptance is mediated by Positive Affect.

**Hypothesis 3:** Basic Need Satisfaction is positively related to Positive Affect.

**Hypothesis 4:** Basic Need Satisfaction moderates the relationship between Self-Compassion and Positive Affect. At low levels of Basic Need Satisfaction, high Self-Compassion is associated with high Positive Affect, but at high levels of Basic Need Satisfaction, Self-Compassion becomes unrelated to Positive Affect.

**Hypothesis 5:** The mediation of Positive Affect on the relation between Self-Compassion and Positive Relations and Social Acceptance is moderated by Basic Need Satisfaction.

## Materials and methods

### Method

In this longitudinal survey, data was collected online (*via* Lime Survey) at two measurement moments with 4 weeks in between.

### Participants

Participants were minimum 18 years old. The participants were recruited *via* social media and the networks of students in psychology at the Open University.

De dataset contained the data of 874 respondents. Of the 874 questionnaires returned, 560 were usable responses, whereas 314 were deemed unusable as data on the second measurement point was missing. They were therefore excluded. As general rule of thumb, the missing responses per variable may range from 0.4 to 10%. Such range is often considered as normal (Hair et al., 2010). Because 314 of 874 respondents were deemed unusable, this exceeds this 10%. Therefore a dropout analysis was done to compare the group that filled in at both measuring moments and the group that dropped out. On the variables used in this study, there were no significant differences between these groups (see Table 1), indicating that this does not influence the results of the analyses. Regarding the survey respondents’ demographics, 34.1% (*N* = 191) are men and 65.9% (*N* = 369) women. The average age is 48 (SD = 13.79). Of these respondents, 32.3% works fulltime (*N* = 181), 45% works part-time (*N* = 252) and 22.7% (*N* = 127) has no paid job. Highest education is high school for 13.8% (*N* = 77), secondary vocational education (MBO) for 12.7% (*N* = 71), studies of applied sciences (HBO) 43.6% (*N* = 244), and studies at research university (WO and higher) 28.7% (*N* = 161).

**TABLE 1:** Drop-out analysis.

*Independent samples test*											
		**Levene’s test for equality of variances**		***t*-Test for equality of means**							
			
		***F***	**Significance**	***t***	**df**	**Significance**		**Mean difference**	**SE difference**	**95% CI of the difference**	
									
						**One-sided *p***	**Two-sided *p***			**Lower**	**Upper**
BNS_T1	Equal variances assumed	0.100	0.752	−1.316	863	0.094	0.188	−0.05129	0.03896	−0.12775	0.02518
	Equal variances not assumed			−1.324	635,207	0.093	0.186	−0.05129	0.03873	−0.12735	0.02477
ZelfCompas_T1	Equal variances assumed	0.179	0.673	−0.617	863	0.269	0.537	−0.02806	0.04548	−0.11733	0.06122
	Equal variances not assumed			−0.616	623,131	0.269	0.538	−0.02806	0.04552	−0.11745	0.06133
PosAffect_T1	Equal variances assumed	0.037	0.848	−0.515	864	0.303	0.606	−0.02283	0.04430	−0.10978	0.06411
	Equal variances not assumed			−0.518	637,670	0.302	0.604	−0.02283	0.04405	−0.10933	0.06366
PRSA_T1	Equal variances assumed	0.272	0.602	−0.044	865	0.483	0.965	−0.00341	0.07826	−0.15702	0.15020
	Equal variances not assumed			−0.044	640,076	0.483	0.965	−0.00341	0.07783	−0.15624	0.14943

T1, time 1; BNS, basic need satisfaction; ZelfCompas, self-compassion; PosAffect, positive affect; PRSA, positive relations and social acceptance.

### Procedure

Participants received information about the study by email. Before entering the study, they were asked to sign the informed consent form. This form stated that participating came without significant risk, was on a voluntary basis and that results would be shared anonymously. This study was approved by the ethics committee of the Open University (U2018/03092/MQF).

### Measures

In addition to the background characteristics (nationality, gender, age, marital status, education level, and work situation), the following questionnaires were included into the survey:

#### Basic need satisfaction

The Basic Psychological Needs Scale (BPNSFS-NL) was used to measure basic need satisfaction. This scale is a self-evaluation questionnaire consisting of 24 items. An example of an item is “I feel capable of reaching my goals.” The respondents answer on a 5-point rating scale. They choose from 1 to 5 to indicate the degree to which the statement is true for them at this point in their life. The scale contains three subscales: autonomy satisfaction, relatedness satisfaction, and competence satisfaction. Reverse items were recode and a sum score was constructed in which high scores represented high satisfaction. Validity reported by Chen et al. (2015) was moderately good. The Cronbach’s alpha’s for autonomy, relatedness, and competence satisfaction were, respectively, 0.86, 0.85, and 0.86. The McDonalds Omega was 0.927.

#### Positive affect

The Positive And Negative Affect Scale (PANAS) has been widely utilized as a self-reported measure of affect in both the community and clinical contexts (Merz et al., 2013). Participants gauge their feelings and respond *via* a questionnaire consisting of 20 items, rated on a 5-point rating scale. Examples of items that are offered in the scale are “excited,” “scared,” or “proud.” Participants are asked to indicate to what extent they feel these emotions at the moment or how they felt over the past week. Sum scores can range from 10 to 50 for both the Positive and Negative Affect with higher scores representing higher levels of Positive/Negative Affect. Reliability and Validity reported by Watson et al. (1988) was moderately good. For the Positive Affect Scale, the Cronbach’s alpha coefficient was 0.84. The McDonalds Omega for the Positive Affect Scale was 0.843.

#### Self-compassion

To measure self-compassion, the Dutch version (Neff and Tóth-Király, 2022) of the Self-Compassion Scale (Neff, 2003) was used. The SCS-NL-R is a self-reporting questionnaire that consists of 26 items, divided over six subscales. Three subscales describe positive attitudes like: self-kindness, shared human experience, and mindfulness, e.g., “When something upsets me I try to keep my emotions in balance.” The other six subscales describe negative attitudes: self-judgment, isolation, and over-identification. An example of this is “When I fail at something important to me I become consumed by feelings of inadequacy.” The respondents are asked to answer on a 7-point rating scale, that goes from 1 (rarely or never) to 7 (almost always) to indicate how often they behave in the indicated manner. Total SCS scores evidenced good internal reliability (Cronbach’s α = 0.92), as did the six subscales with Cronbach’s α ranging from 0.75 to 0.81 (Neff, 2003). In the current study the sum score of all subscales was used to measure self-compassion. The Cronbach’s alpha was 0.92 and the McDonalds Omega was 0.93.

#### Positive relations and social acceptance

To measure positive relations and social acceptance, the Dutch version of the Mental Health Continuum Short Form (MHC-SF) was used (Lamers et al., 2011). The MHC-SF is a self-reporting measure that consists of 14 items divided over three subscales. The items that are used in this research are the ones measuring positive relations (item 11) and social acceptance (item 7). A sum score is made of these two items. An example of an item is “In the past month, how often did you feel you had warm and trusted relations with others?” The respondents answer on a 6-point rating scale going from “never” to “every day.” The MHC-SF has a good reliability with a Cronbach’s α = 0.89 (Lamers et al., 2011). The McDonalds Omega for the two items was 0.58.

### Data analysis

**FIGURE 1:**
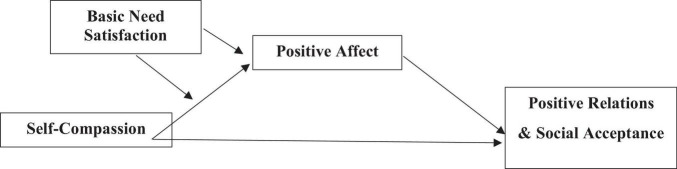
Schematic representation of the conceptual model.

Descriptive statistics, correlation, moderation, and mediating analysis were performed in SPSS version 28. Confirmatory factor analysis (CFA) was conducted to examine the fit of the measurement models for the different constructs. For the analyses structural equation modeling (SEM) with maximum likelihood estimation (AMOS 24) was used. To assess model fit, a number of fit indices were used (Byrne, 2010), Chi-square test (χ^2^), root-mean-square errors of approximation (RMSEA ≤ 0.08), the normed fit index (NFI ≥ 0.90), normed comparative fit index (CFI ≥ 0.90), and the Tucker–Lewis index (TLI ≥ 0.90). To test the mediation of positive affect on the relationship between self-compassion and positive relations and social acceptance hierarchical regression analysis was conducted. The same analysis was used to test the moderation effect of basic need satisfaction on the relationship between self-compassion and positive relations and social acceptance mediated by positive affect. First, a mediation analysis was run using the PROCESS macro in SPSS (Hayes, 2017) to estimate the direct and indirect effects of self-compassion on positive relations and social acceptance through positive affect (Model 4). Second, a moderated mediation analysis was run using the PROCESS macro in SPSS (Hayes, 2017) to estimate the direct and indirect effects of self-compassion on positive relations and social acceptance through positive affect, as moderated by basic need satisfaction (Model 7). The significance of the direct and indirect effects was evaluated by means of 5000 Bootstrap samples to create bias-corrected confidence intervals (CIs; 95%). For the mediation analysis three questionnaires of the 560 were automatically deleted by the software due to missing data in estimating the full model. For the moderated mediation analysis eight questionnaires of the 560 were automatically deleted due to missing data. Gender, age and employment were included as covariates in the model. See Figure 1 for a representation of the research model.

## Results

### Descriptive statistics and correlation analysis

Table 2 shows the descriptive statistics and the Pearson product-moment correlation analysis for self-compassion measured at T1, basic need satisfaction at T2, positive affect at T2 and positive relations, and social acceptance at T2. All the variables were significantly correlated with each other. Positive relations and social acceptance was positively related to positive affect (*r* = 0.46, *p* < 0.01), and positive affect in its turn was positively related to basic need satisfaction (*r* = 0.61, *p* < 0.01) and self-compassion (*r* = 0.46, *p* < 0.01). Self-compassion was positively related to basic need satisfaction (*r* = 0.68, *p* < 0.01) and positive relations and social acceptance (*r* = 0.42, *p* < 0.01). Basic need satisfaction was positively related to positive relations and social acceptance (*r* = 0.52, *p* < 0.01).

**TABLE 2:** Descriptive statistics and correlations.

Variable	*M*	SD	1	2	3
1. Self-compassion T1	3.435	0.639			
2. Basic need satisfaction T2	4.139	0.562	0.676**		
3. Positive affect T2	3.572	0.638	0.458**	0.605**	
4. Positive relations and social acceptance T2	4.448	1.061	0.418**	0.520**	0.455**

**p < 0.01. T1, time 1; T2, time 2.

### Measurement model tests

For self-compassion a second order measurement model was tested first order factors representing the six subscales of the construct loaded on a second order latent factor representing the construct self-compassion. Each item significantly loaded on the subscale it represented. The fit indices of this measurement model [χ^2^(284) = 865.77, NFI = 0.92, CFI = 0.94, TLI = 0.94, RMSEA = 0.05] indicated a good fit. Equally, for basic need satisfaction a second order measurement model was tested first order factors representing the three subscales of the construct loaded on a second order latent factor representing the construct basic need satisfaction. Each item loaded significantly on the subscale it represented. The fit indices of this measurement model [χ^2^(240) = 824.15, NFI = 0.91, CFI = 0.94, TLI = 0.93, RMSEA = 0.05] indicated a good fit. Finally, a measurement model was tested for positive affect. This model included one latent factor, representing positive affect. The fit indices of this measurement model [χ^2^(32) = 152.91, NFI = 0.94, CFI = 0.95, TLI = 0.93, RMSEA = 0.07] indicated a good fit.

### Mediation analysis

Evidence from the estimation of model 4 suggested a direct effect of self-compassion on positive affect (β = 0.47, *p* < 0.01), as shown in Table 3. The results of the mediation analysis showed both self-compassion (β = 0.30, *p* < 0.01) and positive affect (β = 0.33, *p* < 0.01) had a positive effect on positive relations and social acceptance. Furthermore it showed a mediation effect of positive affect on the relation between self-compassion and positive relations and social acceptance (Index 0.15, SE = 0.03, LLCI = 0.11, ULCI = 0.20).

**TABLE 3:** Direct effects.

Variables	Effect	SE	*z*	*p* <	95% CI
Self-compassion T1 → positive affect T2	0.47	0.04	0.46	0.01	[0.39, 0.54]
Self-compassion T1 → positive relations and social acceptance T2	0.30	0.04	0.53	0.01	[0.22, 0.38]
Positive affect T2 → positive relations and social acceptance T2	0.33	0.04	0.53	0.01	[0.25, 0.41]
Basic need satisfaction T2 → positive affect T2	0.51	0.05	0.62	0.01	[0.41, 0.61]

N = 557.

### Moderated mediation analysis

Evidence from the estimation of model 7 suggested a direct effect of basic need satisfaction on positive affect (β = 0.51, *p* < 0.01), The model also suggested that the moderation effect of basic need satisfaction on the mediation of positive affect between self-compassion and positive relations and social acceptance was very close to significant (Index −0.02, SE = 0.01, LLCI = −0.04, ULCI = 0.00). When basic needs were low, this indirect effect was significant (BNS = −1.00, Mediator Index = 0.06, SE = 0.02, LLCI = 0.02, ULCI = 0.10). As expected, basic need satisfaction significantly moderated the effect of self-compassion on positive affect such that for low basic need satisfaction, self-compassion had a significant positive relation with positive affect (β = 0.18, *p* < 0.05). When basic need satisfaction is high, the relation between self-compassion and positive affect became insignificant (β = 0.05, *p* = 0.37).

**FIGURE 2:**
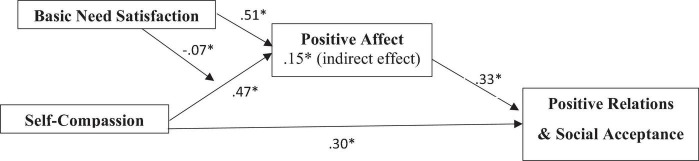
The model with estimates. *p < 0.05.

Results of the full model estimation are illustrated in Figure 2.

## Discussion

The present research tested a model including two individual level factors – self-compassion and basic need satisfaction – which may contribute to build the high-quality relations that are required for psychological safety. Hypotheses 1 and 2 are supported. The positive relation effect of self-compassion on positive affect is in line with previous research (Kreemers et al., 2018). Self-compassion also leads to more positive relations and social acceptance. Although the relationship with positive relations and social acceptance has not been studied as such, previous research does show that self-compassion promotes empathy. Nurses with a higher level of self-compassion showed significantly higher empathy scores (Savieto et al., 2019). The positive effect of self-compassion on positive relations and social acceptance had a similar magnitude than that of positive affect (0.30 vs. 0.33). The second hypothesis referring to the mediation of positive affect on the relationship between self-compassion and positive relations and social acceptance has not been investigated before. However, because self-compassion leads to positive affect, and according to research (Davidson et al., 1990; Fredrickson, 2001) positive affect stimulates us to engage with other people, it makes sense that this positive emotion mediates this relationship between self-compassion and positive relations and social acceptance.

Hypotheses 3 and 4 are also supported. The results showed a significant positive relationship between basic need satisfaction and positive affect (H3), which is also in line with previous research (Stanley et al., 2021). Hypothesis 4 stating that basic need satisfaction moderates the relationship between self-compassion and positive affect was also supported. There is however no previous research into this moderation effect. Results on this moderation showed that when basic need satisfaction was low, self-compassion was more strongly related to positive affect, compared to when satisfaction was average. When basic need satisfaction was high, the relation between self-compassion and positive affect became insignificant. This finding is in line with expectations that self-compassion would be more effective in case of low basic need satisfaction. Neff describes self-compassion as being touched by one’s own suffering (Neff, 2004). In case of low basic need satisfaction, there would be more suffering, than when basic need satisfaction is high. Moreover, when your basic needs satisfaction is high, there is no suffering and self-compassion becomes irrelevant. Finally, hypothesis 5 refers to the moderated mediation which turns out very close to significant. The moderated mediation analysis suggested that basic need satisfaction moderates the mediation of positive affect in the relationship between self-compassion and positive relations and social acceptance when basic needs are low. There was no significant moderated mediation when basic need satisfaction was high. Again, it is likely that self-compassion is only relevant when basic need satisfaction is low because this potentially leads to suffering. It also explains why the overall moderated mediation model is only close to significant, because it depends on whether basic needs have been satisfied or not.

### Theoretical and practical implications

Compared to prior research into psychological safety, this study has added value because it applies an individual perspective through the inclusion of two factors at the individual level: self-compassion and basic need satisfaction. Many previous studies have focused on what people need to do in connection with others to create psychological safety and less on what they need to do in connection with themselves and on the individual characteristics that advance the development of relationships conducive to psychological safety. Frazier et al. (2017) did a meta-review into the antecedents of psychological safety and found much previous research that focused on interpersonal aspects. For example, in their meta-analysis of individual-level psychological safety they found 30 significant positive correlations with psychological safety for positive leader relations, 26 for work design characteristics and 24 for supportive work context. For the personality variables, they found only six for proactive personality, eight for emotional stability, and six for learning orientation. Even though the focus is more on the individual in those studies, they focus on more stable characteristics. None of the antecedents in this meta-review said anything about what individuals need from themselves. In this study we suggest that before people can build this psychologically safe environment, they need a positive emotion that is created by either basic need satisfaction, or self-compassion in case when basic need satisfaction is low. There is partly an individual responsibility in basic need satisfaction. As research shows we can use self-compassion to fill our own basic need of autonomy, connection, and competence (Moè and Katz, 2020), we can take action ourselves whenever we experience a threat to our basic need. However, there is also a responsibility of the leader and organization to create the circumstances where people can fill these needs. For example, Deci et al. (2001) found that managerial autonomy support promotes the satisfaction of all three needs. In the meta-review by Frazier et al. (2017), work design characteristics were shown to be significant antecedents. More specific, autonomy, interdependence and supportive work context were identified as significant antecedents of psychological safety on a group-level. Also practically, focus to build psychological safety has thus far mainly been on dyadic and group relations. This research suggests that this approach can successfully be supplemented by an additional, individualistic, perspective. This is an important addition that will impact possible training and coaching needs in organizations. It also makes the role of the organization more clear in that they should provide the circumstances where people get enough opportunity to fulfill their basic needs of autonomy, connection and competence. Even with plenty opportunity for employees to satisfy their needs, experiencing a threat against their basic needs at some point is inevitable. Therefore an extra step in an organization is to provide the opportunity for employees to further develop their level of self-compassion. For example, Shapiro et al. (2007) found that training in Mindfulness-Based Stress Reduction (MBSR) resulted in a significant increase in self-compassion. Different workplace components like personal factors, contemplative training and leadership styles have been identified that can foster self-compassion (Lefebvre et al., 2020). Banker and Bhal (2020) identify different organizational pressures and enablers that affect the level of compassion. It broadens the area of attention when developing interventions to tackle a lack of psychological safety in an organization, and might even impact what organizations look for in people when they recruit.

### Limitations and further research

As a limitation, our study was based on self-reports, which is subject to common method bias (CMB) (Podsakoff et al., 2003). Although it looks like there are different views about CMB, Jordan and Troth (2020) argued that the debate is not so much about whether CMB exists but about the extent to which this phenomenon affects research findings. Strategies as suggested by Jordan and Troth to minimize the effects of CMB that have been taken in this study are a good research information coversheet and set of instructions, and the use of reverse coded questions. The number of people who dropped out of the study between the two measuring points is another possible limitation. A dropout analysis did not indicate significant changes between people who dropped out and those that filled in the second questionnaire (see Table 1). Drop-out analyses showed that there were not systematically more men or women who dropped out, neither was there a significant difference in dropout for education or age. Another limitation is that the relationship between self-compassion, basic need satisfaction, positive affect, and positive relations and social acceptance between people was studied by using samples from people in the Netherlands and Belgium, which might affect the general applicability of these findings to other cultures (Frazier et al., 2017). Also the samples might not be fully representative for the entire Dutch and Belgian population because more higher educated people took part and samples were taken only through online media. However, education did not seem to have a significant impact on the model.

In this study the focus was on positive relations as an indicator of the impact of the individual process on psychological safety. However, positive relations is not the only antecedent that has been found to impact psychological safety. Frazier et al. (2017) found several other antecedents that were not included in this research. Newman et al. (2017) suggest that even though they found theories linking psychological safety to several antecedents and outcomes, these theories do not provide a detailed and holistic understanding of the underlying processes and mechanisms. The model presented in the current study provides information on the underlying process that supports an individual to build those positive relations. It suggests that people need to feel that their basic needs are satisfied in order to experience the positive emotion that is needed to start building psychological safety. It also suggests that when basic needs are not satisfied, self-compassion can be used to install that positive emotion, which in turn will advance the positive relations one needs to build psychological safety. Future research can expand this model by looking into the underlying process that follows this individual one. Using teams in the sample instead of separate individuals and measuring psychological safety as a dependent variable can provide a better understanding of the subsequent process of creating a psychologically safe team.

## Data availability statement

The raw data supporting the conclusions of this article will be made available by the authors, without undue reservation.

## Ethics statement

The studies involving human participants were reviewed and approved by U2018/03092/MQF Open University, Netherlands. The patients/participants provided their written informed consent to participate in this study.

## Author contributions

LW-S, JV, AB, and NJ contributed to the conception and design of the study. NJ organized the database. LW-S performed the statistical analysis, and wrote the first draft, and sections of the manuscript. All authors contributed to the manuscript revision, read, and approved the submitted version.

## References

[B1] Arch J. J. Brown K. W. Dean D. J. Landy L. N. Brown K. D. Laudenslager M. L. (2014). Self-compassion training modulates alpha-amylase, heart rate variability, and subjective responses to social evaluative threat in women. *Psychoneuroendocrinology* 42 49–58. 10.1016/j.psyneuen.2013.12.018 24636501 PMC3985278

[B2] Banker D. V. Bhal K. T. (2020). Understanding compassion from practicing managers’ perspective: Vicious and virtuous forces in business organizations. *Glob. Bus. Rev.* 21 262–278. 10.1177/0972150917749279

[B3] Bartholomew K. J. Ntoumanis N. Ryan R. M. Bosch J. A. Thøgersen-Ntoumani C. (2011). Self-determination theory and diminished functioning: The role of interpersonal control and psychological need thwarting. *Pers. Soc. Psychol. Bull.* 37 1459–1473. 10.1177/0146167211413125 21700794

[B4] Bartholomew K. J. Ntoumanis N. Thogersen-Ntoumani C. (2009). A review of controlling motivational strategies from a self-determination theory perspective: Implications for sports coaches. *Int. Rev. Sport Exerc. Psychol.* 2 215–233. 10.1080/17509840903235330

[B5] Buruck G. Wendsche J. Melzer M. Strobel A. Dörfel D. (2014). Acute psychosocial stress and emotion regulation skills modulate empathic reactions to pain in others. *Front. Psychol.* 5:517. 10.3389/fpsyg.2014.00517 24910626 PMC4039014

[B6] Byrne B. M. (2010). *Structural Equation Modeling with AMOS: Basic Concepts, Applications, and Programming.* New York, NY: Routledge/Taylor and Francis Group

[B7] Carmeli A. Brueller D. Dutton J. E. (2009). Learning behaviours in the workplace: The role of high-quality interpersonal relationships and psychological safety. *Syst. Res. Behav. Sci.* 26 81–98. 10.1002/sres.932

[B8] Chen B. Vansteenkiste M. Beyers W. Boone L. Deci E. L. Van der Kaap-Deeder J. (2015). Basic psychological need satisfaction, need frustration, and need strength across four cultures. *Motiv. Emot.* 39 216–236. 10.1007/s11031-014-9450-1

[B9] Davidson R. J. Ekman P. Saron C. D. Senulis J. A. Friesen W. V. (1990). Approach-withdrawal and cerebral asymmetry: Emotional expression and brain physiology: I. *J. Pers. Soc. Psychol.* 58:330. 10.1037/0022-3514.58.2.330 2319445

[B10] Deci E. L. Ryan R. M. Gagné M. Leone D. R. Usunov J. Kornazheva B. P. (2001). Need satisfaction, motivation, and well-being in the work organizations of a former eastern bloc country: A cross-cultural study of self-determination. *Pers. Soc. Psychol. Bull.* 27 930–942. 10.1177/0146167201278002

[B11] Diedrich A. Grant M. Hofmann S. G. Hiller W. Berking M. (2014). Self-compassion as an emotion regulation strategy in major depressive disorder. *Behav. Res. Ther.* 58 43–51. 10.1016/j.brat.2014.05.006 24929927

[B12] Edmondson A. (1999). Psychological safety and learning behavior in work teams. *Adm. Sci. Q.* 44 350–383. 10.2307/2666999

[B13] Edmondson A. (2021). Available online at: https://www.psychologytoday.com/us/blog/the-fearless-organization/202109/psychological-safety-is-not-hygiene-factor (accessed February 10, 2022).

[B14] Edmondson A. C. (2002). *Managing the Risk of Learning: Psychological Safety in Work Teams.* Cambridge, MA: Division of Research, Harvard Business School, 255–275. 10.1002/9780470696712.ch13

[B15] Frazier M. L. Fainshmidt S. Klinger R. L. Pezeshkan A. Vracheva V. (2017). Psychological safety: A meta-analytic review and extension. *Pers. Psychol.* 70 113–165. 10.1111/peps.12183

[B16] Fredrickson B. L. (2001). The role of positive emotions in positive psychology: The broaden-and-build theory of positive emotions. *Am. Psychol.* 56:218. 10.1037/0003-066X.56.3.218 11315248 PMC3122271

[B17] Ghorbani N. Watson P. J. Chen Z. Norballa F. (2012). Self-compassion in Iranian Muslims: Relationships with integrative self-knowledge, mental health, and religious orientation. *Int. J. Psychol. Relig.* 22 106–118. 10.1080/10508619.2011.638601

[B18] Hair J. F. Black W. C. Babin B. J. Anderson R. E. (2010). *Multivariate Data Analysis: International Version.* New Jersey, NJ: Pearson.

[B19] Hayes A. F. (2017). *Introduction to Mediation, Moderation, and Conditional Process Analysis: A Regression-Based Approach.* New York, NY: Guilford Press.

[B20] Hebles M. Trincado-Munoz F. Ortega K. (2022). Stress and turnover intentions within healthcare teams: The mediating role of psychological safety, and the moderating effect of COVID-19 worry and supervisor support. *Front. Psychol.* 12:758438. 10.3389/fpsyg.2021.758438 35173646 PMC8841584

[B21] Jordan P. J. Troth A. C. (2020). Common method bias in applied settings: The dilemma of researching in organizations. *Aust. J. Manag.* 45 3–14. 10.1177/0312896219871976

[B22] Kotera Y. Van Gordon W. (2021). Effects of self-compassion training on work-related well-being: A systematic review. *Front. Psychol.* 12:630798. 10.3389/fpsyg.2021.630798 33967896 PMC8102699

[B23] Kreemers L. M. van Hooft E. A. van Vianen A. E. (2018). Dealing with negative job search experiences: The beneficial role of self-compassion for job seekers’ affective responses. *J. Vocat. Behav.* 106 165–179. 10.1016/j.jvb.2018.02.001

[B24] Lakhan R. Agrawal A. Sharma M. (2020). Prevalence of depression, anxiety, and stress during COVID-19 pandemic. *J. Neurosci. Rural Pract.* 11 519–525. 10.1055/s-0040-1716442 33144785 PMC7595780

[B25] Lamers S. M. Westerhof G. J. Bohlmeijer E. T. ten Klooster P. M. Keyes C. L. (2011). Evaluating the psychometric properties of the mental health continuum-short form (MHC-SF). *J. Clin. Psychol.* 67 99–110. 10.1002/jclp.20741 20973032

[B26] Lathren C. R. Rao S. S. Park J. Bluth K. (2021). Self-compassion and current close interpersonal relationships: A scoping literature review. *Mindfulness* 12 1078–1093. 10.1007/s12671-020-01566-5 35309268 PMC8932676

[B27] Leary M. R. (2010). “Affiliation, acceptance, and belonging,” in *Handbook of Social Psychology*, (5th Edn), (eds) Fiske S. T. Gilbert D. T. Lindzey G. (New York, NY: Wiley), 864–897.

[B28] Lefebvre J. I. Montani F. Courcy F. (2020). Self-compassion and resilience at work: A practice-oriented review. *Adv. Dev. Hum. Resour.* 22 437–452. 10.1177/1523422320949145

[B29] May D. R. Gilson R. L. Harter L. M. (2004). The psychological conditions of meaningfulness, safety and availability and the engagement of the human spirit at work. *J. Occup. Organ. Psychol.* 77 11–37. 10.1348/096317904322915892

[B30] Merz E. L. Malcarne V. L. Roesch S. C. Ko C. M. Emerson M. Roma V. G. (2013). Psychometric properties of Positive and Negative Affect Schedule (PANAS) original and short forms in an African American community sample. *J. Affect. Disord.* 151 942–949. 10.1016/j.jad.2013.08.011 24051099 PMC3934411

[B31] Moè A. Katz I. (2020). Self-compassionate teachers are more autonomy supportive and structuring whereas self-derogating teachers are more controlling and chaotic: The mediating role of need satisfaction and burnout. *Teach. Teach. Educ.* 96 103–173. 10.1016/j.tate.2020.103173

[B32] Moore S. Diener E. Tan K. (2018). “Using multiple methods to more fully understand causal relations: Positive affect enhances social relationships,” in *Handbook of Well-Being*, eds Diener E. Oishi S. Tay L. (Salt Lake City, UT: Noba Scholar), 1–17.

[B33] Neff K. (2003). Self-compassion: An alternative conceptualization of a healthy attitude toward oneself. *Self Identity* 2 85–101. 10.1080/15298860309032

[B34] Neff K. (2004). Self-compassion and psychological well-being. *Constructivism Hum. Sci.* 9:27. 10.1037/e633942013-240

[B35] Neff K. D. Kirkpatrick K. L. Rude S. S. (2007). Self-compassion and adaptive psychological functioning. *J. Res. Pers.* 41 139–154. 10.1016/j.jrp.2006.03.004

[B36] Neff K. D. Tóth-Király I. (2022). “Self-compassion scale (SCS),” in *Handbook of assessment in mindfulness research*, eds Medvedev O. N. Krägeloh C. U. Siegert R. J. Singh N. N. (Cham: Springer International Publishing), 1–22.

[B37] Newman A. Donohue R. Eva N. (2017). Psychological safety: A systematic review of the literature. *Hum. Resour. Manag. Rev.* 27 521–535. 10.1016/j.hrmr.2017.01.001

[B38] Podsakoff P. M. MacKenzie S. B. Lee J. Y. Podsakoff N. P. (2003). Common method biases in behavioral research: A critical review of the literature and recommended remedies. *J. Appl. Psychol.* 88:879. 10.1037/0021-9010.88.5.879 14516251

[B39] Ramsey M. A. Gentzler A. L. (2015). An upward spiral: Bidirectional associations between positive affect and positive aspects of close relationships across the life span. *Dev. Rev.* 36 58–104. 10.1016/j.dr.2015.01.003

[B40] Rigoni D. Demanet J. Sartori G. (2015). Happiness in action: The impact of positive affect on the time of the conscious intention to act. *Front. Psychol.* 6:1307. 10.3389/fpsyg.2015.01307 26388812 PMC4554957

[B41] Ryan R. M. Deci E. L. (2000). Self-determination theory and the facilitation of intrinsic motivation, social development, and well-being. *Am. Psychol.* 55:68. 10.1037/0003-066X.55.1.68 11392867

[B42] Savieto R. M. Mercer S. Matos C. C. P. Leão E. R. (2019). Nurses in the triage of the emergency department: Self-compassion and empathy. *Rev. Lat. Am. Enfermagem* 27:e3151. 31340342 10.1590/1518-8345.3049.3151 PMC6687361

[B43] Schein E. H. Bennis W. G. (1965). *Personal and Organizational Change Through Group Methods: The Laboratory Approach.* New York, NY: Wiley.

[B44] Shain M. Arnold I. GermAnn K. (2012). The road to psychological safety: Legal, scientific, and social foundations for a Canadian National Standard on Psychological Safety in the Workplace. *Bull. Sci. Technol. Soc.* 32 142–162. 10.1177/0270467612455737

[B45] Shapiro S. L. Brown K. W. Biegel G. M. (2007). Teaching self-care to caregivers: Effects of mindfulness-based stress reduction on the mental health of therapists in training. *Train. Educ. Prof. Psychol.* 1:105. 10.1037/1931-3918.1.2.105

[B46] Sheldon K. M. Filak V. (2008). Manipulating autonomy, competence, and relatedness support in a game-learning context: New evidence that all three needs matter. *Br. J. Soc. Psychol.* 47 267–283. 10.1348/014466607X238797 17761025

[B47] Stanley P. J. Schutte N. S. Phillips W. J. (2021). A meta-analytic investigation of the relationship between basic psychological need satisfaction and affect. *J. Posit. Sch. Psychol.* 5 1–16. 10.47602/jpsp.v5i1.210

[B48] Svendsen J. L. Osnes B. Binder P. E. Dundas I. Visted E. Nordby H. (2016). Trait self-compassion reflects emotional flexibility through an association with high vagally mediated heart rate variability. *Mindfulness* 7 1103–1113. 10.1007/s12671-016-0549-1 27642372 PMC5010618

[B49] Watson D. Clark L. A. Tellegen A. (1988). Development and validation of brief measures of positive and negative affect: The PANAS scales. *J. Pers. Soc. Psychol.* 54:1063. 10.1037/0022-3514.54.6.1063 3397865

[B50] Ying Y. W. Han M. (2009). Stress and coping with a professional challenge in entering masters of social work students: The role of self-compassion. *J. Relig. Spiritual. Soc. Work* 28 263–283. 10.1080/15426430903070210

[B51] Zessin U. Dickhäuser O. Garbade S. (2015). The relationship between self-compassion and well-being: A meta-analysis. *Appl. Psychol. Health Well Being* 7 340–364. 10.1111/aphw.12051 26311196

[B52] Zhang Y. Luo X. Che X. Duan W. (2016). Protective effect of self-compassion to emotional response among students with chronic academic stress. *Front. Psychol.* 7:1802. 10.3389/fpsyg.2016.01802 27920736 PMC5118418

